# The Effect of Food Matrix Taken with Probiotics on the Survival of Commercial Probiotics in Simulation of Gastrointestinal Digestion

**DOI:** 10.3390/foods13193135

**Published:** 2024-09-30

**Authors:** Primož Treven, Diana Paveljšek, Bojana Bogovič Matijašić, Petra Mohar Lorbeg

**Affiliations:** University of Ljubljana, Biotechnical Faculty, Department of Animal Science, Institute of Dairy Science and Probiotics, Groblje 3, SI-1230 Domžale, Slovenia

**Keywords:** in vitro digestion, commercial probiotics, model food, food matrix, INFOGEST, probiotic food supplement

## Abstract

The adequate survival of probiotics in the harsh environment of the gastrointestinal (GI) tract plays a crucial role in the expression of their functional properties. The aim of the present study was to evaluate the survival of commercial probiotics during digestion using a standardised INFOGEST 2.0 model extended with three food matrices simulating three scenarios for the consumption of probiotics: on an empty stomach, with juice, or with food (porridge). All eight products matched the bacterial content stated on the label. After simulated digestion, we observed an average decrease in viability of 1.6 log10 colony forming units (CFU) when the product was co-digested with water, a 2.5 log10 CFU decrease in the presence of juice, and a 1.2 log10 CFU decrease in the presence of porridge. The survival rate of the probiotics was statistically higher in the test samples with porridge (91.8%) than in those with juice (79.0%). For two products, the number of lactobacilli and bifidobacteria strains after digestion was less than <3 × 10^5^ CFU, which can be considered insufficient. The present study has shown that the survival of probiotic strains during GI passage depends not only on their ability to withstand these harsh conditions but may also be influenced by the manufacturing process and by the foods consumed together with the probiotics.

## 1. Introduction

According to the definition, probiotics are live microorganisms that are able to survive the gastrointestinal (GI) passage maintaining minimal counts [1]. The adequate survival of probiotic microorganisms in the harsh environment of the GI tract plays a crucial role in the expression of their functional properties [2]. Apart from technological stress during food processing, followed by stress related to stabilisation, packaging, and storage, probiotics are exposed to additional stresses after consumption. The ability of a probiotic to survive in the GI tract depends mainly on its acid, bile and enzyme tolerance. During GI passage, strains must tolerate the presence of pepsin and the low pH of the stomach, the presence of enzymes in the duodenum and the antimicrobial activity of bile salts [3]. In addition, resident GI microbiota as well as co-ingested food microbiota can be another important factor [4]. Foods used as carriers of probiotics or taken simultaneously with probiotics can be considered as one of the main factors influencing the ability of probiotics to survive GI passage and allow the expected performance of probiotics in the GI tract [5,6].

In vivo experiments in animal models or clinical studies to test the survival of probiotics during GI passage are often not feasible due to financial or ethical reasons. The diverse range of in vitro models of digestion spans from very simplistic to very complex setups and can be static or dynamic. While most dynamic models are suitable for simulating the digestion of foods and pharmaceutical products, they are relatively complex and costly to set up and maintain. On the contrary, static in vitro digestion models are simpler but still provide good predictions of in vivo digestion outcomes [7,8].

Using various in vitro models of digestion, many studies have investigated the effects of different food matrices on the ability of particular probiotic strains to survive GI passage [9,10,11,12,13,14,15]. Similarly, the survival of several commercial probiotic formulations was evaluated [3,16,17,18,19,20]. The in vitro digestion models used in these studies differed in terms of the number of stages, the chemical composition and pH of the simulated fluids, and the sequential setup. The variety of methods used makes it difficult to compare these results. Therefore, the standardisation of such tests seems to be an important step for comparable results.

The INFOGEST 2.0 in vitro model of gastrointestinal digestion was developed as a harmonised method for evaluating the fate of food components during the digestion process [7,21]. It is a static, three-step digestion method that uses a constant meal-to-digestion fluid ratio and pH for each digestion step, including the oral, gastric, and intestinal phases. The INFOGEST in vitro model has been widely used in food research [22], including the selection and optimisation of different probiotic strains and probiotic formulations [23,24,25,26,27,28,29].

The aim of the present study was to evaluate the survival of commercial probiotic products using the standardised INFOGEST 2.0 model extended by the inclusion of three food matrices simulating three realistic scenarios of probiotic consumption: on an empty stomach, with juice, or with food (porridge). The effect of GI conditions and food constituents on probiotics’ viability and survival has already been extensively studied. However, here we improved the realistic aspect of the well-standardised approach and showed that the extended model could be an important tool for manufacturers to develop technological processes that ensure good survival and efficacy of commercial probiotic preparations.

## 2. Methods

### 2.1. Experimental Design

**Figure 1:**
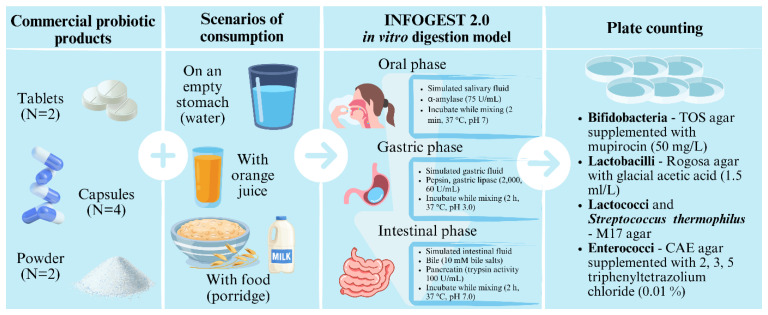
Graphical representation of the experimental design.

We purchased eight probiotic products from a local pharmacy and stored them according to the conditions specified on the labels until analysis (Figure 1). To simulate in vitro GI digestion, we used three different media: sterile purified water, commercially available 100% orange juice (pasteurised), and a meal. For the meal, we mixed 5 g of commercially available finely minced porridge (containing 25% dried fruit) with 15 mL of UHT whole milk (3.5% milk fat) and pasteurised it by heating it above 75 °C for 5 min. We performed viable counts of the labelled bacterial species or groups both before and after the simulated digestion. The tests were performed in triplicates.

### 2.2. Determination of Bacterial Viable Counts

To determine the bacterial viable count prior to simulated digestion, two products in powder form (samples #1 and #5) were prepared for consumption according to the manufacturer’s instructions: the powder from one package was dissolved in 125 mL of sterile distilled water, mixed, and further analysed after one minute. The samples in capsules (samples #2, #3, #4, and #6) or tablets (#7 and #8) were added to buffered peptone water (Merck, Darmstadt, Germany) at a ratio of 1:99 (*w*/*v*). The mixtures were incubated at 37 °C for 30–45 min and vigorously shaken from time to time until the capsule/tablet was completely dissolved. We then serially diluted homogenised samples in ¼ strength Ringer’s solution (Merck, Darmstadt, Germany) and inoculated them into selective media as follows: TOS (Sigma, Darmstadt, Germany) supplemented with mupirocin (50 mg/L) (AppliChem, Darmstadt, Germany) for the enumeration of bifidobacteria, Rogosa agar (Merck, Darmstadt, Germany) with glacial acetic acid (1.5 mL/L) (Merck, Darmstadt, Germany) for the enumeration of lactobacilli, M17 agar (Merck, Darmstadt, Germany) for lactococci and *Streptococcus (S.) thermophilus* enumeration, and CAE agar (HiMedia, Nashik, India) supplemented with 2, 3, 5 triphenyltetrazolium chloride (0.01%) (Merck, Darmstadt, Germany) for the enumeration of enterococci. The plates for enumeration of bifidobacteria and lactobacilli were incubated for 72 h at 37 °C in anaerobic conditions. Enterococci and *S. thermophilus* plates were incubated aerobically at 37 °C for 72 and 48 h, respectively. Lactococci were enumerated after 72 h of aerobic incubation at 30 °C. Since enterococci can also grow on M17 agar at 30 °C, the total count of lactococci in sample #5 which contained both enterococci and lactococci was determined by subtracting the number of presumptive enterococci from the lactococci.

### 2.3. Chemicals and Reagents for In Vitro Digestion

Simulated salivary fluid (SSF), simulated gastric fluid (SGF), and simulated intestinal fluids (SIF) were prepared according to Brodkorb et al. [7]. The chemicals were purchased from Sigma-Aldrich, Steinheim, Germany (CaCl_2_·2H_2_O, MgCl_2_·6H_2_O, KCl), Merck Darmstadt, Germany (KH_2_PO_4_, NaCl), and Honneywell, Seelze, Germany ((NH_4_)_2_CO_3_). The enzymes used were α-amylase from human saliva (Sigma, St. Luis, MO, USA, A1031), pepsin and gastric lipase as “rabbit gastric extract” (RGE) by Lipolytech (Marseille, France), pancreatin from porcine pancreas (Sigma, St. Luis, MO, USA, P1750, 4xUSP), and bovine bile (Sigma-Aldrich, St. Luis, MO, USA, B8631).

### 2.4. In Vitro Digestion

In vitro digestion was performed according to the INFOGEST protocol [7] in aseptic conditions. Briefly, the oral phase was prepared by mixing 2 g of prepared meal, 2 mL of water, or 2 mL of orange juice with 1.75 mL of SSF, 12.5 µL of 0.3 M CaCl_2_·2H_2_O, 0.25 mL of α-amylase from human saliva in water (1500 U/mL), and 0.488 mL of water. During the 2 min incubation, the mixture was manually mixed. Sample #7 was in the form of chewable tablets and was therefore added at the beginning of the oral phase while the remaining seven products were intended to be swallowed without chewing and were therefore added at the end of the oral phase: one whole capsule or one tablet was added together with water to reach 0.5 g. However, the two samples in powder form (#1 and #5) were prepared as described in Chapter 2.2 and 0.5 mL was added in the oral phase. Then, for the gastric phase, 3.75 mL of SGF was added together with 0.1 mL of 0.1 M HCl, 2.5 µL of 0.3 M CaCl_2_·2H_2_O, 0.8 mL of lipase, and pepsin solution in water (lipase 750 U/mL, pepsin 25,000 U/mL). After adjusting the pH to 3.0 ± 0.1, water was added to reach a final volume of 10 mL. The gastric mixture was incubated for two hours with stirring (75 rpm). Finally, the intestinal phase was performed by adding 5.5 mL of SIF, 2.5 mL of porcine pancreatin solution in SIF (8 mg/mL of 4xUSP pancreatin Sigma), 1.25 mL of 160 mM bile salt solution in water, 20 µL of 0.3 M CaCl_2_·2H_2_O, and 75 µL of 1 M NaOH. The pH was adjusted to 7.0 ± 0.1 and water was added to reach a final volume of 20 mL. After incubation at 37 °C for 2 h with gentle agitation (75 rpm), 1 mL of the resulting mixture was used to quantify the number of viable probiotic bacteria, as previously described.

Statistical comparison between samples was performed with GraphPad Prism 5 (Graph Pad software, version 8.4.3) using non-parametric one-way ANOVA (Kruskal–Wallis statistic) and Dunn’s Multiple Comparison Test with an adjustment of *p*-values to account for multiple comparisons. A *p*-value of <0.05 was considered statistically significant.

## 3. Results and Discussion

According to the definition, one of the basic requirements for probiotics is to survive passage through the GI tract [1]. The effect of the food matrix consumed simultaneously with probiotics is often neglected when testing probiotic survival in the GI tract, although it can have a major impact [9,10,11,12,13,14,15]. In order to carry out probiotic survival testing that more closely resembles a realistic situation in the gut and could also be applicable to the industry, we used a standardised in vitro digestion model. We tested three real-life scenarios: when a consumer takes the product on an empty stomach, with commercially available orange juice, or with a meal. As a model meal, we chose commercially available, finely minced porridge prepared with whole milk. We used commercially available foods in order to facilitate the potential standardisation of the procedure since commercially available goods follow Codex Alimentarius standards in all aspects of food production.

Most of the products tested (Table 1) were two- or multi-strain probiotics (6/8), while two of them were single-strain products. The products were in the form of capsules (4/6), powder (2/6), or tablets (2/6). None of the capsules were resistant to gastric juice. All samples contained additional complex sugar ingredients that could affect the survival of the strains in the digestive tract [13,30,31]. Six of them contained inulin or related sugars (fructo-oligosaccharides (FOS) or oligofructose). Interestingly, inulin was listed in one of the labels only, while prebiotics were not mentioned at all.

Two products had a recommended dose of one unit, while the recommended dose for most products was more than one unit (two to three units). To improve the survival and efficiency of the probiotics, the producers include some specific instructions on the labels of the products. Two products (#7 and #8) had no additional instructions except recommended dosage and two products required activation in water for 1 min before consumption (#1 and #5). Hot drinks and alcoholic beverages could significantly affect the survival of probiotics in the GI passage, although some strains may be adapted to survive higher concentrations of ethanol [32] or are adequately protected to withstand high temperatures [33]. Only in the case of samples #2 and #3, the consumers were instructed not to take the product directly with hot drinks, hot food, or alcoholic beverages. Probiotics are often recommended to prevent antibiotic-associated diarrhoea [34]. To achieve the desired effect, probiotics should not be taken at the same time as antibiotics. Accordingly, for four products, users were instructed to take the product 1–3 h before or after taking antibiotics. Although the food matrix can have a major impact on the survival of probiotic strains during GI passage [15], only half of the products included instructions regarding food and probiotic intake. For products #2 and #3, the users were instructed to take the product during a meal, while for #4 and #5, they were instructed to take it on an empty stomach.

**Figure 2:**
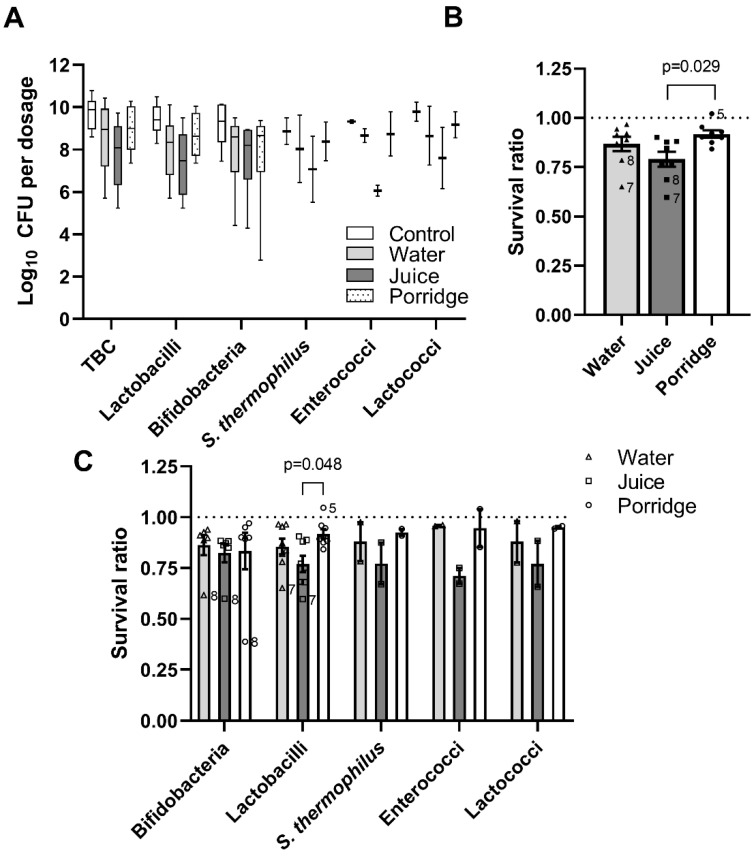
Viability and survival rate of probiotic bacteria within the model food matrix after the simulation of digestion. (**A**) Viability of total bacterial count (TBC) and viability of different groups or species (*S. thermophilus*) in colony-forming units (CFU). (**B**) Survival ratio of bacteria in terms of TBC. (**C**) Survival ratio in terms of individual genera. Each dot represents one probiotic product measured in three biological replicates. The black line represents the mean ± standard error of the mean. Numbers below some data points indicate particularly deviant samples. Numbers below some data points indicate particularly deviant samples.

The appropriate dosage of live probiotic bacteria to provide health benefits to the host is debatable and should be supported by in vivo clinical studies [35]. However, most studies argue that at least 10^6^–10^7^ CFU of probiotic bacteria should reach the colon in a viable state to ensure the expected beneficial effect [36], although some probiotic strains can also be beneficial to the host in a non-viable state [37]. The initial bacterial content in the products was 8.6–10.8 log10 CFU (Figure 2A, Table 2). Most of the products contained an excessive number of viable bacteria. Manufacturers usually add excessive amounts of microbes in probiotic supplements to ensure that the labelled concentration of CFU is reached until the expiration date [38]. All products matched the label claims, although product #8 did not reach the stated CFU number but was still within an acceptable accuracy of 0.5 log10. The quality of the products tested in this study has already been assessed in a previous study using plate counting, matrix-assisted laser desorption ionisation time-of-flight mass spectrometry (MALDI-TOF MS), and species-or subspecies-specific PCR [39]. In the study of Mohar Lorbeg et al., product #6 had an insufficient number of CFU [39]. Based on the present results, it appears that the manufacturer increased the input number of probiotics in the product or improved the manufacturing process to achieve the labelled CFU number. The discrepancy between labelled values and actual measurements of the number of CFU in the products can also be caused by factors such as reduced cultivability and inadequate culture conditions. In addition, relying on culture-dependent methods to evaluate microbes is only effective if all probiotic microorganisms can grow in vitro, which can be challenging in formulations containing multiple species [40].

The variability of survival rates between samples increased considerably after the simulated digestion. When different products were co-digested with water, a similar pattern of survival rates was seen in terms of individual genera. Simulated co-digestion with water led to an average 1.6 log10 CFU decrease in viability. In terms of total bacterial count (TBC), the survival rate of the probiotics co-digested with water was, thus, 87.2% (Figure 2B). An excellent survival rate with a reduction of less than 1 log10 CFU was observed for four products, while a moderate decrease in viability (1–4 log10 CFU) was observed for the other samples. Our results are generally comparable to other similar studies, although some authors even reported no survival after simulated digestion. Aziz, Zaidi, and Tariq [16] performed simulated digestion according to the INFOGEST protocol and observed that about 78% of the products showed no post-gastrointestinal viability. Similarly, Naissinger da Silva, Tagliapietra, Flores, and Pereira Dos Santos Richards [18] reported that only one out of eleven probiotic preparations had a good survival rate, while in four products, the bacterial count after digestion was less than 5.5 log10 CFU. The test of probiotic powders, probiotic capsules, fermented milk, and probiotic-enriched products available in the USA and Canada (N = 29) also found that 24% of products showed excellent survival, 45% showed a reduction of 1–5 log10 CFU, and 31% showed no survival [3]. It should be noted that both studies did not follow the INFOGEST protocol but their own protocols, including simulated gastric digestion at a lower pH.

We observed the highest decrease in viability when the probiotics were digested simultaneously with juice. We observed an average decrease of 2.5 log10 CFU, ranging from 1.1 to 5.3 log10 CFU. Low pH of the food matrix such as orange juice has been previously shown to affect the survival of probiotics during storage as well as in GI digestion [13,14,41]. Although the oral phase in SSF lasted only 2 min, it appears that the initial lower pH of the SSF when mixed with different food matrices (the pH of SSF and water was 8.4; the pH of SSF and juice was 3.8; the pH of SSF and porridge was 6.2) could affect the survival of some probiotic strains during digestion, even though the pH levels of the gastric and intestinal phases were adjusted in all three scenarios. It should be also noted that fruit juices may contain different organic acids that might also affect the survival of probiotics [6]. The lowest decrease in viable counts was seen under the condition of co-digestion with porridge (1.2 log10 CFU), ranging from 0.2 log10 CFU increase to 3.7 log10 CFU decrease in survival. The survival rate of probiotics was statistically higher in the samples with porridge (91.8%) than in those with juice (79.0%). Many studies have shown that fruits and vegetables are also valuable sources of nutrients that make them ideal substrates for the growth of probiotics [6]. Nevertheless, the behaviour of probiotic strains in these food matrices might change during digestion. For example, *B. animalis* subsp. *lactis* Bb-12 showed a good survival rate in orange juice during the 7-day storage but did not survive the subsequent simulation of GI digestion [14]. Tompkins et al. [15] used an in vitro digestion system that simulated the human upper gastrointestinal tract to test the survival of a probiotic product containing four different microbes. The survival of all bacteria in the product was the best when co-digested with cooked oatmeal with milk or when the meal was added to a digestion mixture 30 min after the probiotic. Survival in milk with 1% milk fat and oatmeal–milk gruel was significantly better than in apple juice or in spring water. In line with this Matouskova, Hoova, Rysavka, and Marova [13] observed an increased number of viable cells after co-digestion with porridge. The authors stated that combining mixtures with foods rich in proteins and sugars seems to be the best way to increase the survival of probiotics during digestion. In addition, milk is known to be a suitable environment for probiotic bacteria due to the milk buffering effect that can protect the strains from the harmful effects of the gastric and duodenal environment [12].

Products #7 and #8 deviated from the others with a lower survival rate, especially in water and juice. We observed similar trends when we checked the survival rate of each genus (Figure 2C)—the survival rates of bifidobacteria from sample #8 and lactobacilli from sample #7 were considerably lower compared to the other samples. For example, only 2.7 log10 CFU of bifidobacteria survived the test in the samples with porridge and 5.2 log10 CFU of lactobacilli from sample #7 survived the test in the samples with juice (Table 2). As the labels of these two products did not contain any specific instructions for use (Table 1), the likelihood of consumers receiving an inadequate dose of live probiotic strains is even greater.

The higher variation in the survival rate of lactobacilli is consistent with the fact that, compared to bifidobacteria, lactobacilli represent a very diverse group of microorganisms with different phenotypic characteristics such as the ability to survive under stress conditions [42,43]. In addition, the lactobacilli and enterococci strains from sample #5 showed a slightly increased number of viable bacteria after simulated digestion in porridge. This indicates that the porridge prepared with milk not only provided protection for the bacteria but also served as a source of nutrients for bacterial growth [5]. Interestingly, the manufacturers had given instructions to take product #5 on an empty stomach, but our results showed better survival rates when the product was digested together with the model food. Similarly, compared to co-digestion with water, the survival rate of samples #2 and #3 was not substantially higher in the samples with porridge, even though the manufacturers instructed them to be taken with food.

Besides the inherent limitations of in vitro digestion models, the main limitation of our study was the small number of samples analysed. Nevertheless, we have shown that this approach can be standardised and used to more realistically evaluate the survival of probiotics during passage through the GI tract.

## 4. Conclusions

In the present study, we have adopted the well-known standardised INFOGEST model to include the aspect of real-life conditions. In conclusion, our study has shown that the survival of individual strains during passage through the GI tract depends not only on the ability of the individual strains to withstand these harsh conditions but can also be influenced by the formulation of the product and the foods that are consumed together with the product. As we have observed that some of the probiotic products have not effectively protected the survival of the strains in the GI tract, it would be important for manufacturers to use advanced technologies and to develop a production process that ensures good stability of the products and the survival of the probiotic strains after digestion. The use of gastro-resistant capsules and the microencapsulation of probiotics are common approaches already used by the industry. In addition, we have shown that appropriate instructions for the consumption of the products can help to improve the survival of the strains during GI passage. Finally, the standardised INFOGEST model in combination with three food matrices provides a standardised option for the evaluation of the GI viability of final probiotic products and the evaluation of the instructions for consumers.

## Figures and Tables

**Table 1:** Composition of commercial probiotic products and compliance with the labelled number of CFU ^1^.

Sample	Labelled Strains	Additional Nutritional Ingredients	Instruction for Use	Labelled Number (CFU)	Number Determined by Plate Counting (CFU)
#1	*L. acidophilus* W55, *L. acidophilus* W37, *L. paracasei* W72, *L. rhamnosus* W71, *E. faecium* W54, *L. salivarius* W24, *L. plantarum* W62, *B. bifidum* W23, *B. lactis* W18, *B. longum* W51	corn starch, maltodextrin, inulin, rice protein, FOS, amylase	One-minute activation in water before drinking, 1 h before or after ATB. One to two dosages daily.	5 × 10^9^/sachet	7.2 × 10^9^ CFU/sachet
#2	*L. acidophilus* LA-5, *B. animalis* subsp. *lactis* BB-12	oligofructose	During the meal, 3 h after ATB, not at the same time as hot drinks or alcohol. One to three dosages daily.	2 × 10^9^ CFU/capsule	4.1 × 10^9^ CFU/capsule
#3	*B. animalis* subsp. *lactis* UABla-12, *L. paracasei* UABLpc-04, *B. breve* UaBr-11, *L. gasseri* UALg-05, *L. rhamnosus* UALr-06, *L. rhamnosus* UALr-18, *L. acidophilus* DDS^®^-1, *L. plantarum* UAlp-05, *B. longum* subsp. *longum* UABl-14, *B. bifidum* UABb-10, *L. casei* UALc-03, *L. reuteri* UALre-16, *Lc. lactis* UALl-08, *B. longum* subsp. *infantis* UABi-08	maltodextrin, inulin, water-soluble cellulose	Directly with a meal. Not at the same time as hot food or beverages. Two to three hours before or after taking ATB. One dosage daily.	3 × 10^10^ CFU/capsule	6.2 × 10^10^ CFU/capsule
#4	*L. acidophilus* La-14, *L. plantarum* Lp-115, *L. rhamnosus* Lr-32, *B. breve* BB-03, *L. salivarius* Ls-33, *B. lactis* Bl-04, *L. casei* Lc-11, *L. paracasei* Lpc-37, *S. thermophilus* St-21, *B. longum* Bl-05	FOS, microcrystalline cellulose, ascorbyl palmitate	Between meals or on an empty stomach. One to two dosages daily.	2.5 × 10^10^ CFU/capsule	3.2 × 10^10^ CFU/capsule
#5	*B. animalis* W53, *L. acidophilus* W55, *L. salivarius* W57, *E. faecium* W54, *Lc. lactis* W58, *L. casei* W56	corn starch, maltodextrin, inulin, rice protein, FOS, polydextrose, amylase	One-minute activation in water before drinking. Recommended on an empty stomach. One to two dosages daily.	3 × 10^9^/sachet	8.7 × 10^9^ CFU/sachet
#6	*L. rhamnosus* GG (ATCC 53103)	hydroxypropyl methylcellulose, maltodextrin, gellan gum	With a lot of cold water, 2 h after ATB. One dosage daily.	1 × 10^10^ cells/capsule	1.6 × 10^10^ CFU/capsule
#7	*Limosilactibacillus reuteri* Protectis (DSM 17938)	isomaltose, xylitol	One to two dosages daily.	1 × 10^8^ CFU/tablet	5.7 × 10^8^ CFU/tablet
#8	*L. acidophilus, L. casei, L. plantarum, L. reuteri, L. rhamnosus, B. longum, S. thermophilus*	inulin, cholecalciferol, maltodextrin	One to two dosages daily.	1 × 10^9^ CFU/tablet	4.2 × 10^8^ CFU/tablet *

^1^ CFU—colony forming units; ATB—antibiotic; FOS—fructooligosaccharides; *B.—Bifidobacterium; E.—Enterococcus; L.—Lactobacillus; S.—Streptococcus; Lc.—Lactococcus*. * within 0.5 log_10_ acceptable accuracy.

**Table 2:** Viability of commercial probiotic microorganisms before and after digestion with INFOGEST in vitro model. All concentrations are in log10 CFU (colony forming units) ± standard error of the mean (SEM) per dosage ^1^.

Sample	Food	TBC ^2^		Lactobacilli		Bifidobacteria		Enterococci		Lactococci		*S. thermophilus*	
		Initial	End	Initial	End	Initial	End	Initial	End	Initial	End	Initial	End
#1	Water	9.86 ± 0.02	9.02 ± 0.33	9.44 ± 0.03	7.74 ± 0.40	9.43 ± 0.06	8.74 ± 0.31	9.24 ± 0.02	8.34 ± 0.58	/	/	/	/
	Juice		8.34 ± 0.37		6.43 ± 0.07 *		8.33 ± 0.37		5.81 ± 1.14	/	/	/	/
	Porridge		9.09 ± 0.30		8.33 ± 0.39		8.96 ± 0.28		7.69 ± 0.39	/	/	/	/
#2	Water	9.61 ± 0.06	8.54 ± 0.02	9.34 ± 0.06	7.83 ± 0.08 **	9.27 ± 0.07	8.44 ± 0.04	/	/	/	/	/	/
	Juice		8.46 ± 0.13		8.23 ± 0.14		8.07 ± 0.13	/	/	/	/	/	/
	Porridge		8.65 ± 0.16		8.34 ± 0.22		8.33 ± 0.12	/	/	/	/	/	/
#3	Water	10.49 ± 0.03	10.43 ± 0.08	10.29 ± 0.03	10.13 ± 0.11	10.12 ± 0.10	9.50 ± 0.06	/	/	10.36 ± 0.03	10.43 ± 0.04	/	/
	Juice		9.72 ± 0.18		9.49 ± 0.24		8.92 ± 0.04	/	/		9.72 ± 0.10	/	/
	Porridge		10.27 ± 0.07		10.05 ± 0.04		9.03 ± 0.28	/	/		10.29 ± 0.21	/	/
#4	Water	10.33 ± 0.31	9.55 ± 0.61	9.62 ± 0.40	9.24 ± 0.52	10.13 ± 0.28	9.00 ± 0.61	/	/	/	/	9.49 ± 0.38	9.62 ± 0.28
	Juice		9.33 ± 0.16		8.89 ± 0.17		8.95 ± 0.09	/	/	/	/		8.64 ± 0.26
	Porridge		9.88 ± 0.33		9.49 ± 0.32		9.37 ± 0.21	/	/	/	/		9.29 ± 0.49
#5	Water	9.90 ± 0.13	9.34 ± 0.30	9.38 ± 0.26	9.06 ± 0.30	8.65 ± 0.07	7.77 ± 0.37	9.41 ± 0.03	8.99 ± 0.30	9.34 ± 0.16	7.27 ± na	/	/
	Juice		7.60 ± 0.47		7.07 ± 0.61		7.35 ± 0.45		6.32 ± 0.50		6.16 ± 0.67	/	/
	Porridge		10.12 ± 0.18		9.81 ± 0.17		8.39 ± 0.17		9.78 ± 0.20		8.55 ± na	/	/
#6	Water	10.19 ± 0.05	8.88 ± 0.12	10.19 ± 0.05	8.88 ± 0.12	/	/	/	/	/	/	/	/
	Juice		7.86 ± 0.05 *		7.86 ± 0.05 *	/	/	/	/	/	/	/	/
	Porridge		8.92 ± 0.16		8.92 ± 0.16	/	/	/	/	/	/	/	/
#7	Water	8.75 ± 0.04	5.70 ± 0.07	8.75 ± 0.04	5.70 ± 0.07	/	/	/	/	/	/	/	/
	Juice		5.24 ± 0.06 *		5.24 ± 0.06 *	/	/	/	/	/	/	/	/
	Porridge		7.37 ± 0.52		7.37 ± 0.52	/	/	/	/	/	/	/	/
#8	Water	8.61 ± 0.06	6.76 ± 0.30	8.30 ± 0.08	6.48 ± 0.33	7.46 ± 0.21	4.41 ± na	/	/	/	/	8.24 ± 0.04	6.45 ± 0.28
	Juice		5.92 ± 0.33 *		5.66 ± 0.30 *		4.28 ± na	/	/	/	/		5.52 ± 0.44
	Porridge		7.77 ± 0.16		7.49 ± 0.17		2.78 ± na	/	/	/	/		7.48 ± 0.19

^1^ na—non-available due to parallels below the limit of detection./– not present in the product; *S.—Streptococcus.* ^2^ TBC—total bacterial count, calculated as a sum of all counted microorganisms. Statistical significance (* *p* < 0.05; ** *p* < 0.01) of non-parametric one-way ANOVA (Kruskal–Wallis statistic) and Dunn’s Multiple Comparison Test against initial CFU number, with adjustment of *p*-values to account for multiple comparisons within the experiment (sample).

## Data Availability

The original contributions presented in the study are included in the article, further inquiries can be directed to the corresponding author.
